# Cell Culture Adaptation of Porcine Group A Rotavirus: Advances and Challenges for Vaccine Development

**DOI:** 10.3390/v18070718

**Published:** 2026-06-29

**Authors:** Zhen Zhang, Baihe Ma, Shuhua Liu, Xin Chen, Meiliang Guo, Fanxin Liang, Lianrui Li

**Affiliations:** 1 College of Animal Science and Technology, Tarim University, Alar 843300, China; 10757242138@stumail.taru.edu.cn (Z.Z.); 10757242145@stumail.taru.edu.cn (B.M.); 10757232113@stumail.taru.edu.cn (S.L.); 10757231074@stumail.taru.edu.cn (X.C.); 10757242124@stumail.taru.edu.cn (M.G.); 123456@stumail.taru.edu.cn (F.L.); 2 Tarim Animal Husbandry Science and Technology Key Laboratory of Xinjiang Production and Construction Corps, Alar 843300, China; 3 Tarim Animal Disease Diagnosis and Prevention Engineering Laboratory of Xinjiang Production and Construction Corps, Alar 843300, China

**Keywords:** porcine group A rotavirus, cell culture adaptation, live attenuated vaccine, VP4 mutations, reversion to virulence

## Abstract

Porcine group A rotavirus (PoRVA) is a significant cause of viral diarrhea in piglets, necessitating urgent global implementation of effective control strategies. This review assesses advancements in PoRVA in vitro cultivation and amplification, crucial for PoRVA vaccine development. Traditional PoRVA cultivation commonly employs primary porcine kidney cells or finite cell lines like MA-104, posing well-documented challenges in scalability, production cost, and their ability to recapitulate the natural intestinal microenvironment. Consequently, research has increasingly focused on adapting PoRVA to alternative systems, particularly immortalized porcine cell lines or physiologically relevant porcine intestinal organoids. This adaptation process, involving serial passaging, can induce genomic alterations and virulence attenuation in piglets, essential for generating live attenuated vaccine (LAV) candidates. Modern biotechnological tools, such as reverse genetics and synthetic genomics, have expedited the creation of recombinant PoRVA strains with defined antigenic profiles and enhanced in vitro growth characteristics. However, a significant concern regarding LAV candidates derived from cell culture adaptation is the risk of virulence reversion upon pig back-passage, necessitating thorough safety and genetic stability evaluations. Nevertheless, utilizing stable cell lines or organoid platforms presents a feasible and cost-effective approach for large-scale PoRVA vaccine production. Future research should focus on identifying vaccine candidates that provide broad protection and exceptional safety, with an emphasis on cross-protection against divergent epidemic genotypes, while ensuring the economic feasibility of innovative manufacturing approaches.

## 1. Introduction

Porcine group A rotavirus (PoRVA) is a severe gastrointestinal pathogen affecting newborn piglets, characterized by acute watery diarrhea, dehydration, villous atrophy, and mortality rates exceeding 50% in affected herds [1]. The economic burden of PoRVA is substantial; piglet diarrhea alone has been estimated to cost the Australian pork industry more than AU$7 million annually, with even greater economic losses projected at the global scale [2]. Despite the availability of several commercial vaccines, their efficacy is increasingly compromised by a persistent mismatch between vaccine strains and rapidly evolving field genotypes. In particular, G9 and G4 genotypes have gained dominance in various regions, while the emergence of new reassortants like G9P[23] and G12P[7] poses additional challenges to existing immunity [3,4].

PoRVA belongs to the genus *Rotavirus* within the family Sedoreoviridae and possesses a genome of 11 segments of double-stranded RNA (dsRNA) enclosed in a triple-layered capsid. The outer layer consists of VP7 and VP4, which define G and P genotypes, respectively, and both elicit neutralizing antibodies critical for protective immunity [1,5]. Unlike many other viral pathogens that readily replicate in conventional continuous cell lines, PoRVA exhibits a stringent tropism for mature enterocytes of the small intestine in vivo and, in vitro, requires specific culture conditions that recapitulate the intestinal environment. Historically, PoRVA has been propagated in MA-104 cells (an African green monkey kidney cell line), which have served as the standard substrate for rotavirus isolation and vaccine production [6,7]. More recently, the porcine intestinal epithelial cell line IPEC-J2 and three-dimensional porcine intestinal organoids have been explored as more physiologically relevant models [8,9]. However, each of these systems has notable drawbacks. MA-104 cells, while supporting efficient viral growth, are non-porcine and non-intestinal, which may not fully reflect authentic virus–host interactions. Primary porcine intestinal epithelial cells are difficult to obtain in large quantities and have a limited lifespan, making them impractical for industrial-scale vaccine manufacturing. Moreover, PoRVA employs sophisticated immune evasion mechanisms, such as downregulating swine leukocyte antigen class I (SLA I) expression on the cell surface by degrading β2-microglobulin via the ERAD-proteasome pathway [10]. This evasion complicates the assessment of cell-mediated immune responses and correlates of protection, especially when vaccine candidates are evaluated in simplified culture systems.

The limitations of conventional propagation systems have therefore created a critical bottleneck for the rational development of next-generation PoRVA vaccines. This bottleneck is particularly evident for emerging genotypes that do not adapt easily to standard cell lines or lose immunogenicity upon serial passage. Recent breakthroughs in reverse genetics systems for epidemic strains such as JXAY01 G5P[23] and OSU G5P[7] have enabled the precise engineering of recombinant viruses with improved growth properties, fluorescent reporters, and defined attenuation markers [11,12]. Nevertheless, cell culture-adapted live attenuated vaccine candidates carry the risk of reversion to virulence after back-passage in vivo, which must be carefully evaluated. This review systematically examines the landscape of in vitro propagation systems for PoRVA, including MA-104 cells, IPEC-J2 cells, and porcine intestinal organoids. It evaluates the molecular mechanisms underlying viral adaptation during serial cell culture passage, discusses how modern biotechnological tools (reverse genetics, synthetic biology) accelerate vaccine development, and highlights the safety and cross-protection challenges that remain. By integrating recent advances in cell culture adaptation, structural biology, and immunology, this review aims to inform the design of safe, scalable, and broadly protective PoRVA vaccines that meet the urgent needs of the global swine industry.

## 2. In Vitro Propagation of PoRVA

### 2.1. Primary Porcine Intestinal Epithelial Cells: The Physiological Gold Standard and Its Limitations

Primary porcine intestinal epithelial cells (pIECs) represent the natural target cells of PoRVA infection in vivo and constitute the most physiologically relevant system for studying virus–host interactions during enteric infection. These cells are equipped with key determinants of PoRVA tropism, including terminal sialic acids and histo-blood group antigens, and recapitulate the polarized architecture of the intestinal epithelium, which is essential for understanding viral entry, replication, and immune evasion within the authentic gut microenvironment [9]. Although primary pIECs are invaluable for investigating fundamental aspects of PoRVA biology [13], as Genzel noted in 2015, viral vaccine production predominantly relies on established and designer cell lines that can be scaled up easily in bioreactors, whereas primary cells, by contrast, present significant challenges for such high-volume manufacturing due to their finite proliferative lifespan and stringent culture requirements [14]. Moreover, primary pIECs vary significantly between preparations, leading to batch-to-batch inconsistencies that compromise reproducibility and regulatory compliance. In addition, PoRVA has evolved sophisticated mechanisms to subvert host innate immunity, including the non-structural protein 1 (NSP1) -mediated proteasomal degradation of interferon regulatory factors (IRF3, IRF5, IRF7, and IRF1), which potently suppresses type I and type III interferon responses and complicates the accurate assessment of vaccine-induced immune protection in these primary cultures [15]. To overcome these limitations, researchers have extensively explored alternative propagation platforms, including established cell lines of both porcine and non-porcine origin, as well as advanced three-dimensional intestinal organoid models [16].

### 2.2. Non-Porcine Cell Lines

Among the non-porcine cell lines studied (Table 1), rotavirus entry mechanisms, including the requirement for endosomal acidification, have been extensively investigated using various cell lines, including MA-104 cells [17]. Monkey kidney cells such as MA-104, Vero, and COS have been widely used for porcine rotavirus research and reverse genetics applications [7,18]. The adaptation of porcine group A rotavirus (RVA) to MA-104 cells often results in attenuation of virulence in piglets, which is desirable for live vaccine development. For example, serial passage of field isolates in MA-104 cells can lead to progressive loss of pathogenicity. In one study, complete attenuation was observed after 80 serial passages of three Korean field strains in MA-104 cells; the attenuated viruses remained avirulent in neonatal piglets during five consecutive back-passages, while retaining their antigenicity [4]. However, this study was limited to homologous challenge in a single geographic region with a relatively small sample size, and its cross-protective efficacy against divergent global genotypes remains to be validated. This MA-104-adapted attenuated virus was subsequently used to develop a safe and effective trivalent live vaccine based on three Korean representative epidemic strains (G8P[7] 1741, G9P[23] PRG942, and G5P[7] K71), which provided protection against homologous challenge in pigs. Similarly, a newly isolated G9P[23] strain (RHeN2) was adapted to MA-104 cells, and its inactivated vaccine induced strong neutralizing antibody responses in piglets, showing broad cross-neutralization against P[23] genotype strains [19].

However, adaptation of RVA to MA-104 cells is frequently associated with consistent genomic alterations that drive both enhanced in vitro replication and in vivo attenuation. Comparative genomic studies of multiple human and porcine RVA strains have demonstrated that adaptive mutations cluster predominantly in the VP4 gene, particularly within the hydrophobic VP5 domain. Detailed characterization of key attenuating mutations (e.g., D385N, D393H) and their functional consequences are presented in Section 2.5 and summarized in Table 2. Other monkey kidney cell lines, such as Vero and COS cells, have also been used for RVA adaptation and vaccine production but are likewise associated with genomic changes and attenuation [20,21].

Other cell lines of non-primate origin have also been investigated as possible alternatives. For example, baby hamster kidney (BHK-21) cells have been used to study rotavirus entry mechanisms, but they are not commonly employed for serial propagation of porcine RVA [7,22]. BHK-21 cells have been used mainly for studying virus entry mechanisms rather than for serial propagation. In contrast, human embryonic kidney (HEK293T) cells have been successfully employed in reverse-genetics systems for porcine RVA. Recent advances in reverse genetics have opened new avenues for RVA vaccine development using non-porcine cell lines. Using full genome sequences obtained by nanopore sequencing, a reverse genetics system was established for the OSU G5P[7] strain, enabling recovery of recombinant OSU virus at high titers and construction of a genetically stable recombinant virus expressing the fluorescent UnaG protein [11]. These findings lay a foundation for the rational design of improved live oral or combination vaccines against porcine rotavirus through reverse genetics approaches [11,23].

Notably, unlike enveloped RNA viruses such as porcine epidemic diarrhea virus (PEDV) that frequently accumulate extensive adaptive mutations in the spike gene during heterologous cell adaptation, PoRVA as a segmented double-stranded RNA virus primarily accumulates point mutations during cell culture passage, with no large-scale genomic deletions reported to date. Nevertheless, such mutations may affect the virulence and immunogenicity of the virus in pigs, and therefore careful evaluation is required when developing live attenuated vaccine candidates [4,20,24].

**Table 1:** Comparison of cell culture systems for porcine group A rotavirus (PoRVA) propagation: relevance to vaccine development.

Cell Line	Origin/Cell Type	PoRVA Susceptibility & Adaptation	Key Advantages for Vaccine Development	Key Limitations	References
MA-104	African green monkey kidney, epithelial	High; standard for isolation & serial passage; adaptation often leads to attenuation	Well-characterized; supports high titers [10^5.5^–10^7.5^ TCID50/mL]; widely used for LAV candidates	Non-porcine, non-intestinal; serial passage can induce VP4 mutations that may alter immunogenicity	[4,7,11,20]
Vero	African green monkey kidney, epithelial	Moderate; used for human rotavirus propagation & some vaccine production	Approved for human vaccine manufacturing; scalable	Heterologous; fewer PoRVA adaptation studies compared to MA-104	[7,21]
IPEC-J2	Porcine small intestinal epithelial, homologous	Permissive; supports PoRVA replication & probiotic interaction studies	Physiologically relevant (intestinal origin); preserves natural virus–host interactions; good for immune response & antiviral screening	Finite lifespan in early passages; requires specialized culture conditions	[8,9]
3D porcine intestinal enteroids	Primary porcine intestinal epithelial (stem cell-derived)	Supports virulent & attenuated PoRVA strains; reveals HBGA/SA-dependent replication	Most physiologically relevant; contains all intestinal epithelial cell types; preserves differential replication of attenuated vs. virulent strains	Low throughput; not yet scalable for vaccine production	[9]
LLC-PK1	Porcine kidney epithelial	Permissive; supports PoRVA replication	Polarized phenotype useful for studying entry/release; homologous	Not widely used for vaccine manufacturing; further adaptation studies needed	[20]
ST (swine testis)	Porcine testicular fibroblasts	Permissive; used in commercial rotavirus vaccine production	Homologous; regulatory approval for veterinary vaccines; scalable	Less characterized for PoRVA compared to MA-104	[25,26]
IPIM/iPAM	Immortalized porcine intestinal/alveolar macrophages	Susceptible to several swine viruses; PoRVA susceptibility plausible but unconfirmed	Recapitulate natural target cells (macrophages involved in pathogenesis); useful for studying immune responses	Requires systematic evaluation for PoRVA	[27]
ZMAC-4	Porcine alveolar macrophage (continuous)	Predicted permissive (supports other swine viruses); direct PoRVA adaptation not yet reported	Homologous immune cell type; scalable, GMP-compatible; excellent for LAV production (certified)	No direct PoRVA adaptation studies published to date	[28]

Note: HBGA, histo-blood group antigen; SA, sialic acid; Good Manufacturing Practice (GMP), good manufacturing practice; LAV, live attenuated vaccine.

### 2.3. Homologous Porcine Cell Lines

Notably, several continuous porcine cell lines have shown promise for porcine group A rotavirus (PoRVA) propagation and vaccine development [25,26]. Continuous porcine cell lines derived from alveolar macrophages have been characterized for their susceptibility to various viral pathogens [27,28,29].

Porcine rotavirus A has also been detected in pulmonary tissues of pigs with respiratory disease, suggesting extra-intestinal involvement [30]. However, a porcine macrophage cell line (ZMAC-4) has been shown to support high levels of multiple porcine viral pathogens [28]. While this does not preclude their use in research, it imposes stringent quality control requirements for vaccine manufacturing to ensure adventitious agent safety [31,32].

Other porcine kidney-derived cell lines have also been used for RVA adaptation and replication. Immortalized porcine macrophage cell lines have been characterized for viral vaccine production [25,33], and various continuous cell lines have been evaluated for their susceptibility to multiple porcine enteric pathogens [28,33]. The established susceptibility of swine porcine enterovirus (SPEV) cells to multiple enteric pathogens makes them a promising platform for porcine RVA vaccine development, although direct adaptation studies of porcine RVA to SPEV cells remain limited compared to MA-104 cells.

Another highly valuable porcine cell line is the ST (swine testis) cell line, which has demonstrated particular utility in commercial vaccine production. Studies on rotavirus attenuation have compared virulent and attenuated derivatives in piglets [34], and a strain of porcine rotavirus attenuated by cell culture passages was reported using PK-15 cells [35]. The successful adaptation and regulatory approval of ST cell-based rotavirus vaccines highlight the feasibility of employing homologous porcine cell lines for commercial vaccine manufacturing, offering advantages in physiological relevance compared to heterologous systems.

Porcine small intestinal epithelial cell line IPEC-J2, being a homologous cell line derived from the natural target organ of porcine RVA, offers superior physiological relevance for studying rotavirus infection and host immune responses [36]. The dynamics of a Chinese porcine G9P[23] rotavirus strain were characterized in MA-104 cells and intestines of piglets [37]. Additionally, human rotaviruses of multiple genotypes acquire conserved VP4 mutations during serial passage in cell culture [38]. Using an IPEC-J2-based cell–probiotics–virus platform combined with flow cytometry analysis, recent studies have demonstrated that probiotic bacteria can exert a protective effect on IPEC-J2 cells prior to rotavirus challenge and can also directly reduce viral infection rates [39]. This homologous intestinal epithelial cell system also provides a valuable tool for studying rotavirus-associated alterations in intestinal epithelial lipid metabolism and for screening antiviral compounds, thereby bridging the gap between conventional immortalized cell lines and in vivo animal models.

LLC-PK1 cells, another porcine kidney epithelial cell line, have been widely employed in studies of viral pathogenesis and host–virus interactions. Porcine rotavirus bearing an aberrant gene stemming from an intergenic recombination of the NSP2 and NSP5 genes has been found to be defective and interfering [40], and VP4 mutations identified in a porcine rotavirus adapted to MA-104 cells correlate with reduced virulence [20]. The well-characterized polarized phenotype of LLC-PK1 cells makes them potentially valuable for studying the polarized entry and release of porcine rotavirus, though further adaptation studies are needed to establish their utility for routine RVA vaccine production.

### 2.4. Immortalized Porcine Macrophages

Rotavirus-specific proteins have been detected in murine macrophages in both intestinal and extraintestinal lymphoid tissues [41,42,43]. However, no published studies to date have confirmed productive, sustained PoRVA replication in any immortalized porcine macrophage cell line, including IPIMs, iPAMs, and ZMAC-4. These cell lines have been validated to support efficient replication of several other swine pathogens (e.g., porcine reproductive and respiratory syndrome virus (PRRSV), porcine epidemic diarrhea virus) and possess favorable manufacturing features such as stable karyotypes and GMP compatibility for ZMAC-4. and possess favorable manufacturing features such as stable karyotypes and GMP compatibility for ZMAC-4. Furthermore, the immunological properties of immortalized porcine macrophages, including their SLA-binding profiles, have been characterized in recent studies [44]. Given the documented in vivo tropism of PoRVA to intestinal and alveolar macrophages, these cell lines represent a theoretically promising direction for future research, but their actual suitability for PoRVA propagation and vaccine development requires dedicated experimental validation.

### 2.5. Serial Passage Adaptation and Attenuation Mechanisms

**Figure 1:**
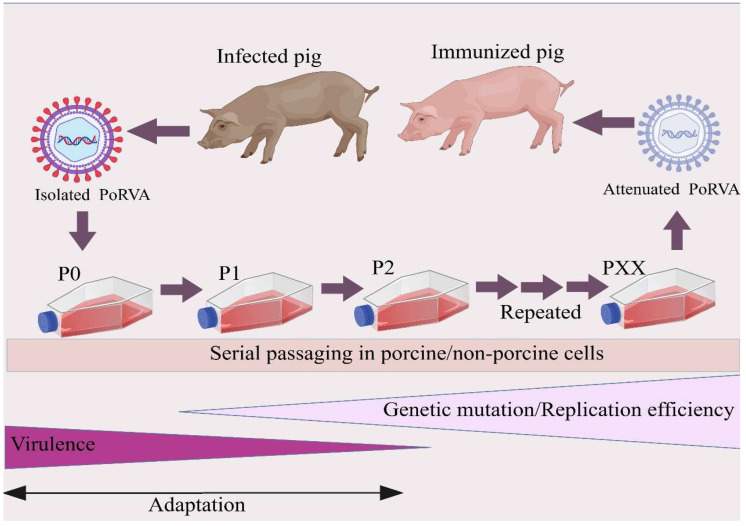
Schematic diagram of serial cell culture adaptation and attenuation of porcine group A rotavirus (PoRVA). The diagram depicts how continuous passaging of wild-type PoRVA in cultured cells accumulates adaptive mutations, enhances in vitro replication efficiency and progressively attenuates in vivo virulence for screening of live attenuated vaccine candidates.

Serial passage is a fundamental technique in virology by which viruses are adapted to grow efficiently in a new host cell type or under specific culture conditions. The process involves the repeated infection of fresh cells with the progeny virus obtained from the previous infection cycle. This iterative process exerts selection pressure on the virus population and favors the survival and replication of virus variants that have advantageous mutations allowing them to more effectively exploit the host cell machinery. A common observation upon serial passage of porcine group A rotavirus (PoRVA) in MA-104 cells, a non-porcine continuous monkey kidney cell line, is a gradual increase in viral titer and replication kinetics upon successive passages. This improved replication efficiency suggests that the virus is increasingly adapting to the new cellular environment [7]. After 10 passages in MA-104 cells, rotavirus isolates typically yield titers between 10^5.5^ and 10^7.5^ TCID_50_/mL, demonstrating robust adaptation to the cell culture setting [7], which may be attributed to the acquisition of conserved mutations in the VP4 gene [37]. These phenotypic adaptations are supported by the accumulation of genetic changes in the viral genome. Such changes can include different types of mutations, including single nucleotide polymorphisms (SNPs), as well as more complex genomic rearrangements such as intragenic and intergenic recombination events. Importantly, the initial genetic mutations that arise during serial passaging are random events, but the selection process that follows is not; the culture conditions favor the survival and replication of variants with specific advantageous mutations, leading to predictable evolutionary pathways depending on the viral strain and cell line used for adaptation. The serial passage procedure for PoRVA adaptation is illustrated in Figure 1.

The adaptation of PoRVA to non-porcine cells frequently results in attenuation or complete loss of virulence in piglets. Serial passage of a pig rotavirus through 15 sequential passages in cell culture produced an attenuated derivative that no longer caused diarrhea in piglets [34]. Comprehensive histopathological and virological characterization revealed that the attenuated virus propagated at a much slower pace in the small intestine, with fewer infected epithelial cells detected at any one time; destruction of enterocytes was never extensive enough to cause marked mucosal changes, and membrane-bound digestive enzymes remained near normal levels [34]. Similarly, a virulent porcine rotavirus isolated from diarrheic piglets in Jiangsu Province was serially passaged in MA-104 cells, with the 90th passage virus almost completely losing its pathogenicity. Notably, serial passaging of porcine rotavirus in cell culture has been shown to yield attenuated strains that remain safe upon consecutive in vivo passages while retaining their antigenic properties [45,46,47]. These findings collectively demonstrate that cell culture adaptation leads to attenuation characterized by a decreased ability to propagate efficiently in enterocytes. Building on these traditional passage-based attenuation strategies, reverse-genetics approaches now enable the rational design of multivalent recombinant strains with precisely defined genotypes; for instance, a recent study successfully generated a trivalent recombinant porcine rotavirus simultaneously expressing VP7 proteins of G4, G5, and G9 genotypes, which conferred broad protective immunity against multiple G-type infections in a mouse model [45]. The successful implementation of such advanced vaccine candidates, however, also relies on the availability of continuously stable and well-characterized production cell substrates to ensure consistent viral growth kinetics and regulatory compliance throughout the manufacturing process [46].

The attenuation observed upon PoRVA adaptation in heterologous cells is closely linked to specific genomic changes, but a potential drawback is that extensive passage may alter the ability of the virus to replicate efficiently in its natural host cells. Unlike enteric coronaviruses such as PEDV that may lose enteric tropism after prolonged passage in heterologous cell lines, PoRVA strains adapted to MA-104 cells generally retain the ability to infect piglets, albeit with significantly reduced pathogenicity. For instance, the 6th passage of a Chinese G9P[23] strain (HN03) in MA-104 cells, when administered to 3-day-old piglets, still caused severe watery diarrhea within 24 h post-inoculation, indicating that relatively early-passage virus maintained considerable virulence [37]. However, the 90th passage of another strain had essentially lost all virulence while retaining immunogenicity, suggesting that there is a passage level at which attenuation is achieved without complete loss of the ability to replicate in the natural host. This trade-off between attenuation and reduced in vivo fitness remains incompletely understood, but likely involves modifications to the viral surface proteins that mediate host cell attachment and entry, particularly VP4 and VP7.

Genomic analysis of serially passaged PoRVA strains confirms that VP4 is the most mutation-prone segment during cell culture adaptation. A landmark comparative study of four human and porcine RVA strains (virulent piglet-passaged strains vs. MA-104-adapted attenuated derivatives) found that 81.2% of nonsynonymous mutations localized to the VP4 gene, with a strong hotspot in the hydrophobic VP5 domain [20]. Key conserved attenuating mutations across strains include D385N and S471H/L, with a strain-specific D393H substitution identified in the attenuated OSU strain. A full list of validated attenuation-associated mutations, along with their phenotypic effects, is provided in Table 2.

The VP4 protein is the viral attachment protein that mediates cell entry and is cleaved by trypsin into VP5 and VP8 fragments, a process essential for infectivity. Given that these hydrophobic region mutations are conserved across multiple strains, it is plausible that they affect the efficiency of membrane fusion or the conformational changes required for cell entry, thereby contributing to the attenuated phenotype in vivo. No large-scale genomic deletions comparable to those observed for enteric coronaviruses such as PEDV during prolonged cell culture adaptation have been reported for PoRVA; instead, attenuation appears to be driven primarily by point mutations in VP4 and, to a lesser extent, in other genes such as NSP4, which is involved in calcium homeostasis and enterotoxin activity.

In addition to point mutations, serial passage can also induce more complex genomic rearrangements, especially when viruses are passaged at high multiplicity of infection. Serial undiluted passage of a porcine rotavirus in MA-104 cells yielded three distinct virus populations, each bearing different rearranged genes [40]. Two populations carried distinct intragenic recombinant NSP3 genes consisting of partial duplications in a head-to-tail orientation without altering the NSP3 open reading frame, and both were viable. The third population carried both an intragenic recombinant NSP3 gene and an intergenic recombinant gene (1647 nucleotides in length) which contained a truncated NSP2 gene inserted into the NSP5 gene at residue 332; this latter population was defective and interfering [40]. These rearranged genomes arise from non-homologous recombination events during viral RNA replication, a process facilitated by the high local concentration of replicating RNA segments within the viroplasm. While such rearrangements are not required for attenuation per se since point mutations suffice, they represent an additional layer of genetic diversity that can emerge under certain passage conditions, though vaccine development efforts typically avoid high-MOI passaging to minimize the generation of defective interfering particles.

While serial passage in MA-104 cells has historically been the standard method for PoRVA adaptation and attenuation, it is a relatively uncontrolled evolutionary process that can lead to a complex and sometimes unpredictable set of genetic changes. Consequently, thorough genomic and phenotypic characterization of the adapted virus at each passage level is crucial to understand the mechanisms of adaptation and possible unintended consequences for its suitability as a vaccine candidate. Recent advances in high-throughput sequencing and reverse genetics have begun to illuminate the molecular basis of PoRVA attenuation. Using complete genome sequences determined via Nanopore sequencing, a robust reverse genetics system was developed for the OSU G5P[7] strain, enabling the recovery of recombinant OSU rotavirus that can be used for the production of improved vaccines targeting the most common cause of porcine rotavirus infections [11,12]. Similarly, a plasmid-based reverse genetics system was established for the NJ2012 G9P[7] strain, generating recombinant reporter viruses expressing fluorescent UnaG and NLuc proteins. Importantly, this system was used to develop a novel trivalent recombinant vaccine against prevalent PoRV epidemic genotypes, with promising immunoprotective effects [45]. These reverse genetics platforms allow researchers to move beyond the random discovery of attenuated strains through serial passage toward a more rational and predictive model for vaccine development, where specific genetic changes identified in passaged viruses such as the D385N and D393H mutations in VP4 can be intentionally introduced into wild-type backgrounds to generate precisely engineered attenuated vaccine candidates [20]. Ultimately, this systems biology approach represents a decisive shift away from empirical attenuation toward a more deliberate and controlled strategy for vaccine design, where specific mutations correlated with desired safety and efficacy outcomes can be incorporated while minimizing extraneous genomic changes (Table 2).

**Table 2:** Key genomic mutations associated with rotavirus (porcine, human, and murine strains) attenuation during serial passage in cell cultures.

Virus Strain (Genotype)	Passaging Cell Line	Passage Level	Genomic Segment	Mutation (Amino Acid Change)	Functional Domain	Phenotypic Effect (Attenuation)	References
OSU G5P[7]	MA-104	43	VP4 (VP5 region)	D393H (unique)	Hydrophobic region (VP4)	Reduced virulence in piglets; no diarrhea after 90 passages	[4,20]
SB1A G5P[7]	MA-104	90	VP4 (VP5), others	Multiple point mutations (incl. D393H-like)	Hydrophobic region	Complete loss of pathogenicity; retained antigenicity	[4]
Gottfried G4P[6]	MA-104	54	VP4	D385N, S471H/L	Hydrophobic region	Attenuation in piglets; conserved across multiple strain pairs	[20,48]
Wa G1P[8] human	MA-104	25	VP4	D385N, S471H/L	Hydrophobic region	Attenuation; used as model for cross-species comparison	[20]
M G3P[8] human	MA-104	43	VP4	D385N	Hydrophobic region	Attenuation	[20]
CDC9 G1P[8] human	Vero	45	VP5 (part of VP4)	A331V and/or D385N	VP5 region	Increased in vitro replication; reduced shedding in neonatal rats	[21,24]
Chinese field strain G9P[23]	MA-104	6 (early)	VP4, others	Not fully characterized—still virulent	—	Severe diarrhea in piglets; insufficient attenuation	[37]
EB (murine rotavirus)	MA-104 → mouse pups	N/A	VP4 & NSP4	VP4 substitutions + NSP4 V37A	NSP4 enterotoxin domain	Reversion to virulence; V37A sufficient to enhance diarrheagenic activity	[49]

Note: Functional validation of the D385N and D393H mutations in VP4 as attenuating determinants has been derived primarily from human rotavirus (Wa, M, CDC-9) and murine rotavirus (EB) models, as indicated in the references. While these findings provide strong mechanistic insights applicable to PoRVA, direct reverse-genetics confirmation in PoRVA backgrounds remains limited. Therefore, the phenotypic effects listed for PoRVA strains are inferred from sequence conservation and in vivo attenuation data, but should be interpreted with this caveat.

### 2.6. Safety and Efficacy of Cell Culture-Adapted Live Attenuated Vaccine Candidates

The use of live attenuated viruses (LAVs) derived from traditional cell culture passage is a promising strategy for the development of effective vaccines against porcine group A rotavirus (PoRVA). LAVs are designed to elicit a robust and long-lasting immune response by mimicking a natural infection without causing severe disease in the host. A common approach to achieve attenuation is to adapt PoRVA to non-porcine cell lines, particularly MA-104 cells derived from monkey kidneys. This process often leads to a reduction in the virulence of the virus in piglets, making these adapted strains attractive candidates for the development of LAVs.

Several examples of cell culture adaptation have been documented, each with unique characteristics that provide insight into the attenuation process. A virulent porcine rotavirus isolate SB-1A G5P[7] serially passaged in MA-104 cells showed progressive attenuation, with the 90th-passage virus almost completely losing its pathogenicity in piglets. Importantly, this highly passaged virus remained safe even after being passed through the alimentary canal of newborn piglets three times consecutively, while retaining its antigenicity [4]. Vaccination with the trivalent vaccine induced no diarrhea during the first two weeks post-vaccination, and challenge of trivalent-vaccinated piglets with homologous virulent strains did not induce diarrhea for two weeks post-challenge [4]. Fecal secretory IgAs specific for each vaccine strain were detected starting at 14 days post-vaccination, and serum virus-neutralizing antibodies were induced by 7 days post-vaccination [4]. No diarrhea was observed in any experimental piglets during five consecutive passages of each vaccine strain. These data collectively indicated that the live attenuated trivalent vaccine was safe and effective at protecting piglets from diarrhea induced by challenge exposure of homologous virulent strains (Table 3).

A critical consideration for PoRVA LAVs derived from cell culture passage is their genetic stability and potential to revert to virulence. In the Korean trivalent vaccine study, no diarrhea was observed in any experimental piglets during five consecutive passages of each vaccine strain in vivo, indicating that the attenuating mutations remained stable under those conditions [4]. However, studies in related rotavirus models have shown that cell-culture-adapted avirulent strains can become diarrheagenic after serial in vivo passages, highlighting the importance of rigorous safety testing. For example, a G5P[23] porcine rotavirus strain was isolated and its pathogenicity was analyzed [49,50,51]. Intraperitoneal injection of recombinant NSP4 proteins confirmed that the aa 37 site is important for its diarrheagenic activity in mice [49]. These observations underscore the necessity of comprehensive genetic stability studies and monitoring for reversion of attenuating mutations during the development of PoRVA LAV candidates.

Furthermore, studies of host–pathogen interactions have shown that effective LAVs must balance attenuation with the ability to stimulate humoral and cellular immune responses. In the gnotobiotic pig model, rotavirus-specific interferon-gamma (IFNγ) producing CD4^+^, CD8^+^, and CD4^+^CD8^+^ T cell responses were examined in pigs infected with a virulent human rotavirus or vaccinated with an attenuated human rotavirus vaccine [41,52]. It was demonstrated that virus-specific intestinal IFNγ producing T cell responses induced by human rotavirus infection and vaccines are correlated with protection against rotavirus diarrhea, highlighting the importance of cellular immune responses in vaccine-induced protection [52]. While the trivalent vaccine developed with 80 passages in MA-104 cells was both safe and effective [4], the 6th passage of a Chinese G9P[23] strain (HN03) in MA-104 cells, when administered to 3-day-old piglets, still caused severe watery diarrhea within 24 h post-inoculation, indicating that relatively early-passage virus maintained considerable virulence and thus insufficient attenuation. Conversely, the 90th passage of another strain had essentially lost all virulence while retaining immunogenicity, suggesting that an optimal passage level exists where attenuation is achieved without compromising the ability to stimulate a protective immune response.

### 2.7. Genetically Engineered and Biotechnologically Enhanced Strains

Recent advances in PoRVA vaccine development have expanded beyond traditional cell culture adaptation approaches. Researchers are now employing targeted reverse genetics techniques and site-directed mutagenesis to generate rationally designed attenuated strains with specific genomic modifications.

Using complete genome sequences determined via Nanopore sequencing, a robust reverse genetics system enabling the recovery of recombinant rOSU rotavirus was developed [11]. Although rOSU grew to high titers (~10^7^ plaque-forming units/mL), its growth kinetics were modestly decreased in comparison to the laboratory-adapted OSU virus. By engineering a fused NSP3-2A-UnaG open reading frame into segment 7 RNA, a genetically stable rOSU virus expressing the fluorescent UnaG protein as a functional separate product was produced [11]. These findings raise the possibility of producing improved live oral porcine rotavirus vaccines through reverse-genetics-based modification or combination vaccines that can express neutralizing antigens for other porcine enteric diseases.

Similarly, a reverse genetics system for the PoRV strain NJ2012 G9P[7] was established, and researchers successfully constructed multivalent recombinant PoRV strains by inserting the VP7 gene of the G4 genotype into the NSP3 gene segment and/or the VP7 gene of the G5 genotype into the NSP1 gene segment within the backbone of the rNJ2012WT strain [45]. These multivalent recombinant viruses efficiently expressed G4 and/or G5 genotype VP7 proteins in infected cells and elicited robust immune responses in mice, with the trivalent recombinant virus simultaneously expressing VP7 proteins from G4, G5, and G9 genotypes conferring passive protection to suckling mice against infections caused by multiple G genotypes of PoRV [45]. In a separate approach using the simian rotavirus SA11 strain as a backbone, a reverse genetics system was established for generating recombinant PoRV vaccine candidates carrying VP4 and VP7 genes [53]. Reverse genetics platforms based on the simian rotavirus SA11 strain have been successfully used to generate recombinant viruses carrying heterologous VP4 genes from diverse species, including pigs [54]. Such systems also enable the simultaneous incorporation of VP4 and VP7 genes into the SA11 backbone, yielding chimeric viruses that can induce robust neutralizing antibody responses in mice [53].

The molecular determinants of successful attenuation have been further elucidated through site-directed mutagenesis studies. Studies on the human rotavirus vaccine CDC9 G1P[8] identified that mutations of wild-type CDC9 P11 at the VP5 region AA331 and AA385, each or in combination, were associated with increased replication in vitro comparable to cell culture-passaged CDC9 P45 [21,24,55]. Neonatal rats infected with the single AA331 or AA385 mutant had reduced viral shedding, comparable to cell-culture-passaged CDC9 P45 [24]. This study was the first to identify molecular signatures that define attenuation of a human rotavirus vaccine, providing great potential for targeted mutation in rotavirus vaccine generation instead of labor-consuming serial passaging in cell culture [24]. In a murine model, a cell culture-adapted avirulent rotavirus EB strain was used to generate site-directed revertants, confirming that a single amino acid substitution at NSP4 aa37 (Val to Ala) is sufficient to enhance diarrheagenic activity [49].

#### AI/ML-Driven Prediction of Adaptive and Attenuating Mutations

Recent advances in artificial intelligence (AI) and machine learning (ML) have provided new computational tools to accelerate PoRVA vaccine development by predicting adaptive mutations and attenuating phenotypes without labor-intensive empirical serial passaging. Genome language models trained on large-scale viral genome datasets can infer the fitness effects of amino acid substitutions and identify mutations that enhance in vitro replication while reducing in vivo virulence. For rotaviruses specifically, ML models have been applied to predict antigenic drift of VP7 and VP4 proteins, map conserved neutralizing epitopes, and optimize viral growth conditions in cell culture. However, to date, no AI/ML models have been specifically trained on PoRVA adaptation and attenuation datasets, and the predictive performance of existing general viral models for PoRVA remains to be experimentally validated. Future integration of multi-omics data from PoRVA passage studies with deep learning models will enable precise prediction of genetically stable attenuated strains, significantly shortening the vaccine development cycle and reducing development costs.

In addition to direct viral genome modification, CRISPR/Cas-based systems have been employed to develop innovative vaccine delivery platforms. An auxotrophic vaccine platform was developed using the CRISPR-Cas9D10A gene editing system to construct *Lactobacillus casei* expressing the major protective antigen VP4 of PoRV [56]. The target gene, alanine racemase (*alr*), in the genome of *L. casei* W56 was knocked out, and a recombinant strain pPG-alr-VP4/Δalr W56 was constructed. Mice orally immunized with this strain exhibited high levels of serum IgG and mucosal secretory immunoglobulin A (SIgA) with neutralizing effects against PoRV [56]. This antibiotic-free LAB platform provides a safer oral vaccine strategy against PoRV infection (Table 4).

Modern biotechnologies are playing an increasingly important role in facilitating and accelerating the development of modified PoRVA strains for research and vaccine purposes. These approaches offer advantages over traditional serial passage methods by allowing more precise control of genetic modifications and reducing the time required to develop vaccine candidates.

The reverse genetics systems described in Section 2.7 represent a major technological leap. In addition to the OSU and NJ2012 systems, a reverse genetics system for the epidemic PoRVA strain JXAY01 [G5P[23]I12R1C1M1A8N1T7E1H1] has been established [12]. Eleven JXAY01 genome segment plasmids were cotransfected with ten complementary SA11 genome plasmids, and 11 monoreassortant strains were successfully rescued. Whole genome sequencing showed 12 amino acid differences between the isolate JXAY01 and the recombinant rJXAY01, but no significant difference in their in vitro replication ability was observed [12].

CRISPR/Cas-based systems have also emerged as a powerful tool, not only for constructing engineered *Lactobacillus* strains (as described in Section 2.7) but also for potential direct editing of the rotavirus genome in the future. The combination of reverse genetics and CRISPR technologies enables a more rational and predictive model for vaccine development.

Innovative cell culture systems have been developed to better mimic the natural intestinal microenvironment. Three-dimensional (3D) porcine intestinal enteroid (PIE) cultures contain all intestinal epithelial cell types identified in vivo and represent a unique physiologically functional model to study rotavirus pathogenesis in vitro [9,58]. Differentiated PIEs infected with multiple virulent and attenuated PoRVA strains demonstrated that differential histo-blood group antigen (HBGA)-rotavirus and sialic acid (SA)-rotavirus interactions determine replication efficacy, showing that 3D enteroids impose more relevant selection pressures on the virus during infection [9,57]. The ability of 3D enteroid systems to maintain differentiated cell populations and authentic tissue architecture provides a microenvironment that closely mirrors natural PoRVA infection, holding promise for future vaccine candidate evaluation [9,50,57,59].

To summarize, modern biotechnologies are transforming the landscape of PoRVA vaccine development. Reverse genetics systems for multiple PoRVA genotypes have enabled rapid construction of recombinant viruses without labor-intensive serial passaging. CRISPR-based strategies have enabled innovative oral vaccine delivery systems. Three-dimensional intestinal organoid cultures offer a more physiologically relevant environment for studying host–pathogen interactions. These technological advances collectively move the field from empirical cell culture passage to a more rational, controlled, and predictive model for vaccine development.

### 2.8. Reversion to Virulence

**Figure 2:**
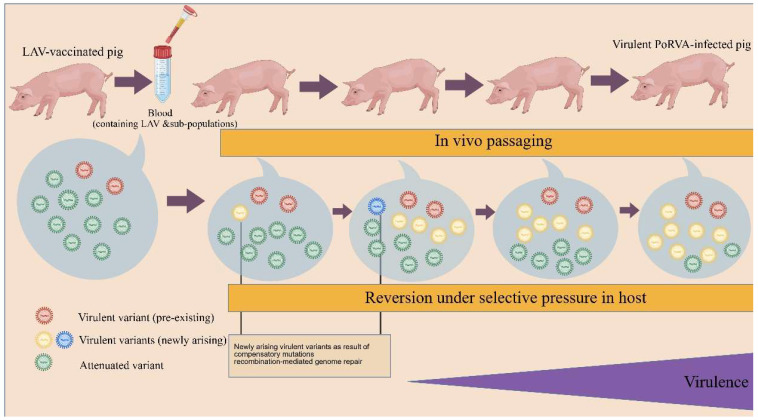
Schematic diagram of virulence reversion of PoRVA live attenuated vaccines (LAVs). The diagram illustrates the generation and enrichment of virulent revertants from attenuated vaccine strains during in vivo replication in piglets, covering both pre-existing low-abundance virulent subpopulations and de novo variants arising under host selective pressure.

A critical safety risk associated with the development of live attenuated vaccines (LAVs) against PoRVA, whether derived by heterologous cell culture adaptation or genetic modification, is the possibility that the attenuated virus may revert to a virulent form upon in vivo passage in piglets. This risk must be thoroughly evaluated before an LAV can be considered safe for widespread use (Figure 2).

Recent advances in genomic surveillance have revealed complex patterns of genetic changes associated with reversion to virulence in rotavirus vaccine candidates. A landmark study in Belgium identified 80 vaccine-derived rotavirus strains among 5125 rotavirus-positive infants with gastroenteritis from 2007 to 2018 [60]. Reconstruction of 30 near-complete vaccine-derived genomes revealed 0–11 mutations per genome, with 88% being nonsynonymous. Several shared amino acid changes pointed to selection of minor variant(s) already present in the vaccine, and some substitutions were identified as true revertants (e.g., F167L on VP4, and I45T on NSP4) that restored sequences towards the unattenuated vaccine precursor strain [60]. This study provided direct evidence that rotavirus vaccine strains can acquire reversions of attenuating mutations during replication in vaccinated hosts.

In the porcine rotavirus context, direct evidence for reversion to virulence has been documented using the murine rotavirus EB model. A cell-culture-adapted avirulent EB strain passaged serially in mouse pups regained virulence, almost consistently acquiring four kinds of amino acid substitutions in VP4 and a substitution at aa 37 (Val to Ala) in NSP4, in addition to gaining the 3′ consensus sequence in NSP1 [49]. The molecular changes occurred with acquisition of virulence in mice and disappeared following passages in cell cultures, demonstrating that the reversion process is reversible and selection-dependent.

Rotavirus can also utilize recombination and reassortment mechanisms to restore virulence. The segmented genome facilitates reassortment between vaccine strains and wild-type field strains, generating novel progeny viruses with unpredictable virulence phenotypes. Whole genome analysis of rare reassortant strains from Zambia revealed that animal-like strains may impact vaccine efficacy and safety through reassortment events [61]. Deep sequencing has shown that seemingly stable attenuated vaccine preparations often contain low-abundance virulent variants that can rapidly become dominant under selective pressure, as evidenced by the Belgian Rotarix study [60].

To address these challenges, multidimensional stability assessment protocols should be leveraged for PoRVA LAV candidates. These include prolonged in vivo passaging (5–8 passages) with comprehensive genomic analysis at each passage [4,49]. For PoRVA, the Korean trivalent vaccine strains showed no diarrhea during five consecutive passages, indicating stability under those conditions [4], but the murine EB study shows reversion may require more passages [49]. Deep sequencing (targeting <1% variant allele frequency), transcriptomics, and functional assays are recommended.

Innovative in vitro systems such as 3D intestinal enteroids can provide early indicators of potential instability [9]. Unlike conventional 2D cell lines, porcine intestinal enteroids contain all intestinal epithelial cell types and support differential replication of virulent versus attenuated strains [9]. Computational modeling using AI/ML trained on rotavirus genome sequences could predict genetic stability, considering factors such as sequence context, protein structure, and specific attenuating mutation positions (e.g., aa385/393 in VP4, aa37 in NSP4) [20,62,63].

Regulatory guidelines from World Health Organization (WHO) and national pharmacopoeias require characterization of vaccine seeds and working cell banks, including tests for adventitious agents, residual cellular DNA tumorigenicity, and genetic stability. For porcine rotavirus vaccines, additional considerations apply regarding the porcine-specific safety profile and reversion potential in the target species.

Ultimately, ensuring the safety of PoRVA LAVs requires an integrated application of advanced in vivo, in vitro, and in silico tools. The successful Korean trivalent vaccine represents an important step forward [4,60], but documented cases of rotavirus vaccine-derived gastroenteritis in humans serve as a cautionary tale [60,64]. For PoRVA specifically, the primary attenuating mutations in the VP4 hydrophobic region (positions 385 and 393) and in NSP4 (position 37) should be specifically monitored during in vivo safety studies [20,49]. Rationally designed deletion mutations [rather than point mutations alone] may offer greater genetic stability, and introducing multiple independent attenuating mutations via reverse genetics could reduce the probability of reversion through single compensatory events.

## 3. Discussion and Conclusions

### 3.1. Summary of Core Findings

This review systematically evaluated the current landscape of PoRVA in vitro propagation systems and their implications for vaccine development. The core findings can be summarized as follows: (1) Heterologous cell lines represented by MA-104 remain the gold standard for PoRVA isolation, serial passage attenuation, and current commercial vaccine production, with well-characterized protocols and proven scalability, but their non-intestinal, non-porcine origin drives adaptive mutations primarily in the VP4 hydrophobic region, which may alter viral immunogenicity and carry reversion risk. (2) Homologous porcine cell lines (IPEC-J2, ST, LLC-PK1) offer improved physiological relevance, with ST cells already approved for commercial vaccine use, but most lack systematic adaptation studies for PoRVA. (3) Immortalized porcine macrophage cell lines (ZMAC-4, IPIM, iPAM) are theoretically promising homologous substrates, but no published data have confirmed PoRVA replication in these cells, requiring dedicated experimental validation. (4) Three-dimensional porcine intestinal organoids represent the most physiologically relevant in vitro model for evaluating viral tropism and attenuation, but low throughput and high cost currently limit their use in large-scale vaccine manufacturing. (5) Reverse genetics, multivalent recombinant design, and CRISPR-based delivery systems have transformed PoRVA vaccine development from empirical screening to rational design, enabling precise attenuation and broad cross-protection. (6) Reversion to virulence remains the primary safety concern for live attenuated vaccines, requiring comprehensive evaluation combining in vivo passaging, deep sequencing, and computational prediction.

### 3.2. Balanced Evaluation of Propagation Platforms for Vaccine Development

From a practical vaccine manufacturing perspective, each propagation platform has distinct advantages and limitations, which must be weighed against production scale, cost, regulatory compliance, and vaccine performance requirements.

MA-104 cells remain the most mature and cost-effective platform for early-stage vaccine development and industrial production. They support high viral titers (10^5.5^–10^7.5^ TCID_50_/mL), have well-established GMP production protocols, and have a long track record of regulatory approval for veterinary vaccines. However, vaccine strains adapted to MA-104 cells carry VP4 mutations that may reduce cross-protective efficacy against field strains, and long-term genetic stability in vivo requires continuous monitoring.

Homologous porcine cell lines (ST, IPEC-J2) represent a balanced choice between physiological relevance and manufacturability. ST cells have already been used in commercial rotavirus vaccines, with proven regulatory acceptability and scalability, and their porcine origin may better preserve viral antigenic epitopes compared to heterologous cells. IPEC-J2 cells are ideal for preclinical evaluation of viral pathogenesis and immune interactions, but their limited lifespan in early passages and specialized culture requirements increase production costs, making them more suitable for research than large-scale manufacturing.

Three-dimensional intestinal organoids are the most predictive preclinical model for evaluating attenuation, immunogenicity, and reversion risk, as they recapitulate the natural intestinal epithelial microenvironment. However, they are currently not scalable for commercial vaccine production, with high culture costs, low throughput, and complex quality control requirements. Their primary value lies in preclinical candidate screening and mechanism studies, rather than as a production substrate.

Immortalized macrophage cell lines such as ZMAC-4 have notable potential if PoRVA susceptibility is confirmed. Their GMP certification, scalable suspension culture adaptability, and homologous immune cell origin make them attractive candidates for next-generation live attenuated vaccines, but their practical application awaits systematic experimental validation.

From a cost perspective, MA-104 and Vero cells have the lowest production costs due to simple culture conditions and high titer yields. Homologous porcine cell lines have moderately higher costs, while 3D organoids have the highest cost per unit virus, currently limiting their use to early research stages.

### 3.3. Priority Directions for Future Research

Based on the current gaps in PoRVA vaccine research, the following priority directions are proposed for future work:

Systematic evaluation of homologous cell line susceptibility: Conduct dedicated studies to characterize PoRVA replication kinetics, genetic stability, and immunogenicity retention in immortalized porcine macrophage cell lines (ZMAC-4, IPIM, iPAM) and other understudied homologous cell lines, to validate their suitability as vaccine production substrates.

Rational design of genetically stable attenuated strains: Leverage reverse genetics to introduce multiple independent attenuating mutations (e.g., VP4 D385N + NSP4 V37A) into epidemic PoRVA genotypes, to minimize reversion risk while retaining immunogenicity. Develop multivalent vaccine candidates covering the dominant global genotypes (G4, G5, G8, G9, P[6], P[7], P[23]) to address genotype mismatch issues.

Establishment of standardized preclinical evaluation models: Optimize 3D porcine intestinal organoid culture systems to improve throughput, and establish standardized protocols for evaluating vaccine candidate attenuation, immunogenicity, and reversion risk using organoid models, to improve the predictability of preclinical studies.

Development of PoRVA-specific AI prediction models: Curate a dedicated PoRVA adaptation and attenuation genome dataset, train specialized machine learning models to predict attenuating mutations and genetic stability, and accelerate vaccine candidate screening.

Optimization of low-cost manufacturing processes: Adapt high-performing cell lines to serum-free, chemically defined suspension culture conditions, optimize bioreactor production parameters, and reduce vaccine manufacturing costs to improve economic feasibility for global application.

### 3.4. Limitations of This Review

This review has several limitations that should be acknowledged. First, the available literature on PoRVA cell culture adaptation is heavily biased toward MA-104 cells, with relatively few studies on homologous porcine cell lines and organoid systems, limiting the depth of comparative analysis. Second, most of the published vaccine efficacy data come from small-scale animal experiments in single geographic regions, with limited large-scale field trial data, making it difficult to fully evaluate real-world vaccine performance. Third, many of the advanced biotechnologies discussed (e.g., AI/ML prediction, multivalent reverse genetics vaccines) have only been validated in murine models or in vitro systems, with limited data in the natural porcine host, and their translational potential remains to be confirmed. Finally, this review focuses primarily on live attenuated vaccines, with less detailed discussion of inactivated vaccines, subunit vaccines, and mRNA vaccine platforms, which also represent important directions for future PoRVA vaccine development.

## 4. Future Perspectives: Integrating Vaccine Design and Advanced Manufacturing for Porcine Group A Rotavirus

The future of effective control of porcine group A rotavirus (PoRVA)-associated diarrhea depends crucially on the successful development and deployment of safe, effective and affordable vaccines. As this review has detailed, the use of continuous cell lines for vaccine production is the most viable way forward, offering dramatic advantages over primary porcine intestinal epithelial cells or primary macrophages in terms of scalability, lot-to-lot consistency, cost-effectiveness and animal welfare considerations. The ultimate goal is to combine the rational design of exceptionally safe live attenuated or inactivated vaccine candidates with modern, robust production platforms. While significant challenges remain notably ensuring long-term genetic stability to prevent reversion to virulence, avoiding unwanted reassortment with co-circulating field strains, and developing vaccines that provide broad cross-protection against the increasing genetic diversity of PoRVA genotypes [G4, G5, G8, G9 and P[6], P[7], P[13], P[23]] recent breakthroughs in production technology are paving the way.

A significant advance in the adaptation of PoRVA-permissive continuous cell lines (e.g., MA-104, Vero, and immortalized porcine macrophage lines such as ZMAC-4) to serum-free, chemically defined media and suspension cultures represents an important milestone. This advance not only reduces production costs and batch-to-batch variability but is also a critical step towards meeting the stringent good manufacturing practice (GMP) requirements for commercial veterinary vaccine production. By combining these innovative manufacturing methods with increasingly sophisticated vaccine development strategies including reverse-genetics-based stabilization of attenuating mutations (such as those in the VP4 hydrophobic region), CRISPR-Cas-engineered delivery systems, and the use of three-dimensional intestinal enteroids for more predictive pre-production evaluation the field is moving closer to what has long been sought: a globally accessible, commercially viable, and safe porcine rotavirus vaccine that can ultimately control this economically devastating disease in pig herds worldwide.

## Figures and Tables

**Table 3:** Safety and efficacy of live attenuated PoRVA vaccine candidates derived from cell culture adaptation.

Vaccine Candidate (Strain/Genotype)	Attenuation Method (Cell Line, Passages)	Animal Model	Safety Outcome	Efficacy Outcome (Protection)	Reversion-to- Virulence Tested?	References
Trivalent LAV G8P[7] 1741 + G9P[23] PRG942 + G5P[7] K71	MA-104, 80 passages	Neonatal piglets	No diarrhea for 2 weeks post-vaccination; safe over 5 consecutive in vivo passages	Full protection against homologous virulent strains; fecal sIgA & serum neutralizing antibodies induced	Yes (5 passages—stable)	[4]
Monovalent SB1A G5P[7]	MA-104, 90 passages	Piglets	Almost complete loss of pathogenicity; no diarrhea after high passage	Retained antigenicity; immunogenic	Yes (3 passages—no reversion)	[4]
OSU G5P[7]	MA-104, 43 passages	Piglets	Attenuated; no severe disease	Used as backbone for reverse-genetics vaccine candidates	Not directly tested but mutations stable	[11,20]
RHeN2 G9P[23] (inactivated)	MA-104 adapted	Piglets	No safety concerns (inactivated vaccine)	Cross-neutralization against P[23] strains	Not applicable (inactivated)	[19]
Gottfried G4P[6]	MA-104, 54 passages	Gnotobiotic piglets	Reduced diarrhea; slower intestinal replication	Partial protection; used as model for attenuation mechanisms	Not reported	[20,34]
HN03 G9P[23]	MA-104, passage 6	3-day-old piglets	Still caused severe watery diarrhea within 24 h	Not sufficiently attenuated—not a vaccine candidate	Not tested (virulent)	[37]

Note: LAV, live attenuated vaccine; sIgA, secretory immunoglobulin A.

**Table 4:** Advanced biotechnological strategies for PoRVA vaccine development.

Technology Platform	Target Strain/System	Genetic Modification or Approach	Key Outcome	Implication for PoRVA Vaccine Development	References
3D porcine intestinal enteroids	Virulent vs. attenuated PoRVA strains	Differentiated enteroids recapitulate in vivo tropism	Differential replication of PoRVA strains based on HBGA/SA interactions	More predictive preclinical evaluation of attenuation & stability	[9,57]
Reverse genetics (RG)	OSU G5P[7]	Insertion of UnaG fluorescent protein into NSP3 segment	Genetically stable recombinant virus expressing UnaG; high titers (~10^7^ PFU/mL)	Enables rational design of live oral vaccines & combination vectors	[11]
AI/ML-based prediction	Rotavirus genome sequences	Genome language model for adaptation prediction	Framework to predict attenuating mutations & stability	Accelerates identification of genetically stable vaccine candidates	[22]
Site-directed mutagenesis	Human CDC-9 (model for porcine)	Mutations A331V and/or D385N in VP5	Replication comparable to cell-culture-adapted virus; reduced shedding in rats	Identifies minimal attenuation signatures → avoids laborious serial passage	[24]
RG + multivalent design	NJ2012 G9P[7]	Insertion of G4 VP7 into NSP3 and G5 VP7 into NSP1	Trivalent recombinant virus expressing VP7 of G4, G5, G9; passive protection in suckling mice	Broad cross-protection against multiple G genotypes	[44,45]
RG (simian SA11 backbone)	Heterologous expression	Cloning of porcine VP4 & VP7 into SA11	Chimeric viruses induce neutralizing antibodies in mice	Rapid generation of vaccine candidates without extensive passaging	[52,53,54]
CRISPR-Cas9D10A	*Lactobacillus casei* (oral vaccine delivery)	Knockout of *alr* gene; insertion of PoRV VP4	Recombinant *L. casei* induces serum IgG & mucosal SIgA with neutralizing activity	Safe, antibiotic-resistance-free oral vaccine platform	[55]

## Data Availability

No new data were generated for this review article. Data sharing is not applicable to this article.

## References

[1] Martella V., Pratelli A., Greco G., Tempesta M., Ferrari M., Losio M.N., Buonavoglia C. (2001). Genomic characterization of porcine rotaviruses in Italy. Clin. Diagn. Lab. Immunol..

[2] Genz B., Gerszon J., Pollock Y., Gleeson B., Shankar R., Sellars M.J., Moser R.J. (2023). Detection and genetic diversity of porcine rotavirus A, B and C in eastern Australian piggeries. Aust. Vet. J..

[3] Monteagudo L.V., Benito A.A., Lázaro-Gaspar S., Arnal J.L., Martin-Jurado D., Menjon R., Quílez J. (2022). Occurrence of Rotavirus A Genotypes and Other Enteric Pathogens in Diarrheic Suckling Piglets from Spanish Swine Farms. Animals.

[4] Park J.G., Alfajaro M.M., Cho E.H., Kim J.Y., Soliman M., Baek Y.B., Park C.H., Lee J.H., Son K.Y., Cho K.O. (2019). Development of a live attenuated trivalent porcine rotavirus A vaccine against disease caused by recent strains most prevalent in South Korea. Vet. Res..

[5] Pedersen K., Goecke N.B., Canuti M., Ryt-Hansen P., Weber N.R., Cortey M., Larsen L.E. (2026). Genetic and antigenic diversity of rotavirus A in faeces from Danish pigs. Virus Evol..

[6] Saif L.J., Terrett L.A., Miller K.L., Cross R.F. (1988). Serial propagation of porcine group C rotavirus (pararotavirus) in a continuous cell line and characterization of the passaged virus. J. Clin. Microbiol..

[7] Otto P.H., Reetz J., Eichhorn W., Herbst W., Elschner M.C. (2015). Isolation and propagation of the animal rotaviruses in MA-104 cells--30 years of practical experience. J. Virol. Methods.

[8] Schierack P., Nordhoff M., Pollmann M., Weyrauch K.D., Amasheh S., Lodemann U., Jores J., Tachu B., Kleta S., Blikslager A. (2006). Characterization of a porcine intestinal epithelial cell line for in vitro studies of microbial pathogenesis in swine. Histochem. Cell Biol..

[9] Guo Y., Candelero-Rueda R.A., Saif L.J., Vlasova A.N. (2021). Infection of porcine small intestinal enteroids with human and pig rotavirus A strains reveals contrasting roles for histo-blood group antigens and terminal sialic acids. PLoS Pathog..

[10] Jing Z., Wu L., Pan Y., Zhang L., Zhang X., Shi D., Shi H., Chen J., Ji Z., Zhang J. (2024). Rotavirus infection inhibits SLA-I expression on the cell surface by degrading β2 M via ERAD-proteasome pathway. Vet. Microbiol..

[11] Snyder A.J., Agbemabiese C.A., Patton J.T. (2024). Production of OSU G5P[7] Porcine Rotavirus Expressing a Fluorescent Reporter via Reverse Genetics. Viruses.

[12] Liu C., Wei H., Zhang X., Wu W., Shen Z., Luo F., Deng S. (2025). Establishment of a reverse genetics system for an epidemic strain of porcine rotavirus JXAY01 type G5P[23]I12. Front. Vet. Sci..

[13] Cui T., Theuns S., Desmarets L.M.B., Xie J., De Gryse G.M.A., Yang B., Van den Broeck W., Nauwynck H.J. (2018). Establishment of porcine enterocyte/myofibroblast co-cultures for the growth of porcine rota- and coronaviruses. Sci. Rep..

[14] Genzel Y. (2015). Designing cell lines for viral vaccine production: Where do we stand?. Biotechnol. J..

[15] Iaconis G., Jackson B., Childs K., Boyce M., Goodbourn S., Blake N., Iturriza-Gomara M., Seago J. (2021). Rotavirus NSP1 Inhibits Type I and Type III Interferon Induction. Viruses.

[16] Rai V., Rajak K.K., Kiran, Fayaz A., Karki M., Einstein C., Bhatt M., Kumar A., Yadav A.K., Deb R., Yadav A.K., Rajkhowa S., Malik Y.S. (2022). Cell Culture System for Porcine Virus Isolation and Propagation. Protocols for the Diagnosis of Pig Viral Diseases.

[17] de Sautu M., Leistner C., Kirchhausen T., Jenni S., Harrison S.C. (2026). Mechanism of membrane perforation in rotavirus cell entry. bioRxiv.

[18] Zhu Y., Sullender M.E., Campbell D.E., Wang L., Lee S., Kawagishi T., Hou G., Dizdarevic A., Jais P.H., Baldridge M.T. (2024). CRISPR/Cas9 screens identify key host factors that enhance rotavirus reverse genetics efficacy and vaccine production. npj Vaccines.

[19] Wang Z., Huang W., Yan G., Tian Y., Wang C., Mao X., Sun M., Zhou L., Yu C., Xia H. (2025). Isolation, Genomic Characterization, and Immunogenicity Evaluation of a G9P[23] Porcine Rotavirus Strain. Vet. Sci..

[20] Guo Y., Wentworth D.E., Stucker K.M., Halpin R.A., Lam H.C., Marthaler D., Saif L.J., Vlasova A.N. (2020). Amino Acid Substitutions in Positions 385 and 393 of the Hydrophobic Region of VP4 May Be Associated with Rotavirus Attenuation and Cell Culture Adaptation. Viruses.

[21] Resch T.K., Wang Y., Moon S., Jiang B. (2020). Serial Passaging of the Human Rotavirus CDC-9 Strain in Cell Culture Leads to Attenuation: Characterization from In Vitro and In Vivo Studies. J. Virol..

[22] Ciarlet M., Crawford S.E., Cheng E., Blutt S.E., Rice D.A., Bergelson J.M., Estes M.K. (2002). VLA-2 (α2β1) integrin promotes rotavirus entry into cells but is not necessary for rotavirus attachment. J. Virol..

[23] Wang J., Qin S., Li K., Yin X., Sun D., Chang J. (2025). Rotavirus Reverse Genetics Systems and Oral Vaccine Delivery Vectors for Mucosal Vaccination. Microorganisms.

[24] Bessey T.K., Wang Y., Moon S.-S., Sanchez-Tacuba L., Jaïs P.H., Greenberg H.B., Jiang B. (2025). Mutations of two amino acids in VP5 mediate the attenuation of human rotavirus vaccine: Evidence from in vitro and in vivo studies. J. Virol..

[25] Moiseenko D., Chernyshev R., Kamalova N., Gavrilova V., Igolkin A. (2025). Evolution of Porcine Virus Isolation: Guidelines for Practical Laboratory Application. Microorganisms.

[26] Welter M.W. (1998). Adaptation and serial passage of bovine coronavirus in an established diploid swine testicular cell line and subsequent development of a modified live vaccine. Adv. Exp. Med. Biol..

[27] Diep N.V., Hayakawa-Sugaya Y., Ishikawa S., Kawaguchi H., Suda Y., Esaki M., Okuya K., Ozawa M. (2024). Establishment of an Immortalized Porcine Alveolar Macrophage Cell Line That Supports Efficient Replication of Porcine Reproductive and Respiratory Syndrome Viruses. Pathogens.

[28] Portugal R., Goatley L.C., Husmann R., Zuckermann F.A., Dixon L.K. (2020). A porcine macrophage cell line that supports high levels of replication of OURT88/3, an attenuated strain of African swine fever virus. Emerg. Microbes Infect..

[29] Masujin K., Kitamura T., Kameyama K.-I., Okadera K., Nishi T., Takenouchi T., Kitani H., Kokuho T. (2021). An immortalized porcine macrophage cell line competent for the isolation of African swine fever virus. Sci. Rep..

[30] Nelsen A., Lager K.M., Stasko J., Nelson E., Lin C.M., Hause B.M. (2022). Identification of Pulmonary Infections with Porcine Rotavirus A in Pigs with Respiratory Disease. Front. Vet. Sci..

[31] Wessman S.J. (2006). Vaccine cell substrates: Bovine and porcine virus considerations. Dev. Biol..

[32] Khan A.S. (2010). Testing considerations for novel cell substrates: A regulatory perspective. PDA J. Pharm. Sci. Technol..

[33] Meloni D., Franzoni G., Oggiano A. (2022). Cell Lines for the Development of African Swine Fever Virus Vaccine Candidates: An Update. Vaccines.

[34] Tzipori S., Unicomb L., Bishop R., Montenaro J., Vaelioja L.M. (1989). Studies on attenuation of rotavirus. A comparison in piglets between virulent virus and its attenuated derivative. Arch. Virol..

[35] McNulty M.S., Curran W.L., Allan G.M., McFerran J.B. (1978). Synthesis of coreless, probably defective virus particles in cell cultures infected with rotaviruses. Arch. Virol..

[36] Liu F., Li G., Wen K., Bui T., Cao D., Zhang Y., Yuan L. (2010). Porcine small intestinal epithelial cell line [IPEC-J2] of rotavirus infection as a new model for the study of innate immune responses to rotaviruses and probiotics. Viral Immunol..

[37] Wang Z., Lv C., Xu X., Li X., Yao Y., Gao X., Sun Z., Wang Y., Sun Y., Xiao Y. (2018). The dynamics of a Chinese porcine G9P[23] rotavirus production in MA-104 cells and intestines of 3-day-old piglets. J. Vet. Med. Sci..

[38] Carter M.H., Gribble J., Diller J.R., Denison M.R., Mirza S.A., Chappell J.D., Halasa N.B., Ogden K.M. (2024). Human Rotaviruses of Multiple Genotypes Acquire Conserved VP4 Mutations during Serial Passage. Viruses.

[39] Leblanc D., Raymond Y., Lemay M.J., Champagne C.P., Brassard J. (2022). Effect of probiotic bacteria on porcine rotavirus OSU infection of porcine intestinal epithelial IPEC-J2 cells. Arch. Virol..

[40] Cao D., Barro M., Hoshino Y. (2008). Porcine rotavirus bearing an aberrant gene stemming from an intergenic recombination of the NSP2 and NSP5 genes is defective and interfering. J. Virol..

[41] Clarke E., Desselberger U. (2015). Correlates of protection against human rotavirus disease and the factors influencing protection in low-income settings. Mucosal Immunol..

[42] Takenouchi T., Masujin K., Miyazaki A., Suzuki S., Takagi M., Kokuho T., Uenishi H. (2022). Isolation and immortalization of macrophages derived from fetal porcine small intestine and their susceptibility to porcine viral pathogen infections. Front. Vet. Sci..

[43] Brown K.A., Offit P.A. (1998). Rotavirus-specific proteins are detected in murine macrophages in both intestinal and extraintestinal lymphoid tissues. Microb. Pathog..

[44] Van Chanh Le Q., Le T.M., Cho H.S., Kim W.I., Hong K., Song H., Kim J.H., Park C. (2018). Analysis of peptide-SLA binding by establishing immortalized porcine alveolar macrophage cells with different SLA class II haplotypes. Vet. Res..

[45] Cheng X., Deng H., Bian X., Wang J., Wang C., Han N., Zhou J., Zhu X., Zhang X., Yang X. (2026). Simultaneous expression of three G genotypes of VP7 proteins in a recombinant porcine rotavirus confers protective immunity against multiple rotavirus infections. J. Virol..

[46] Rai A., Pruitt S., Ramirez-Medina E., Vuono E.A., Silva E., Velazquez-Salinas L., Carrillo C., Borca M.V., Gladue D.P. (2020). Identification of a Continuously Stable and Commercially Available Cell Line for the Identification of Infectious African Swine Fever Virus in Clinical Samples. Viruses.

[47] Vlasova A.N., Amimo J.O., Saif L.J. (2017). Porcine Rotaviruses: Epidemiology, Immune Responses and Control Strategies. Viruses.

[48] Tsugawa T., Tsutsumi H. (2016). Genomic changes detected after serial passages in cell culture of virulent human G1P[8] rotaviruses. Infect. Genet. Evol..

[49] Tsugawa T., Tatsumi M., Tsutsumi H. (2014). Virulence-associated genome mutations of murine rotavirus identified by alternating serial passages in mice and cell cultures. J. Virol..

[50] Saxena K., Blutt S.E., Ettayebi K., Zeng X.L., Broughman J.R., Crawford S.E., Karandikar U.C., Sastri N.P., Conner M.E., Opekun A.R. (2015). Human Intestinal Enteroids: A New Model To Study Human Rotavirus Infection, Host Restriction, and Pathophysiology. J. Virol..

[51] Gao L., Shen H., Zhao S., Chen S., Zhu P., Lin W., Chen F. (2023). Isolation and Pathogenicity Analysis of a G5P[23] Porcine Rotavirus Strain. Viruses.

[52] Yuan L., Wen K., Azevedo M.S., Gonzalez A.M., Zhang W., Saif L.J. (2008). Virus-specific intestinal IFN-gamma producing T cell responses induced by human rotavirus infection and vaccines are correlated with protection against rotavirus diarrhea in gnotobiotic pigs. Vaccine.

[53] Kanai Y., Onishi M., Kawagishi T., Pannacha P., Nurdin J.A., Nouda R., Yamasaki M., Lusiany T., Khamrin P., Okitsu S. (2020). Reverse Genetics Approach for Developing Rotavirus Vaccine Candidates Carrying VP4 and VP7 Genes Cloned from Clinical Isolates of Human Rotavirus. J. Virol..

[54] Falkenhagen A., Patzina-Mehling C., Gadicherla A.K., Strydom A., O’Neill H.G., Johne R. (2020). Generation of Simian Rotavirus Reassortants with VP4- and VP7-Encoding Genome Segments from Human Strains Circulating in Africa Using Reverse Genetics. Viruses.

[55] Jenni S., Li Z., Wang Y., Bessey T., Salgado E.N., Schmidt A.G., Greenberg H.B., Jiang B., Harrison S.C. (2022). Rotavirus VP4 Epitope of a Broadly Neutralizing Human Antibody Defined by Its Structure Bound with an Attenuated-Strain Virion. J. Virol..

[56] Zhang H., Zhao H., Zhao Y., Sui L., Li F., Zhang H., Li J., Jiang Y., Cui W., Ding G. (2022). Auxotrophic *Lactobacillus* Expressing Porcine Rotavirus VP4 Constructed Using CRISPR-Cas9D10A System Induces Effective Immunity in Mice. Vaccines.

[57] Guo Y., Raev S., Kick M.K., Raque M., Saif L.J., Vlasova A.N. (2022). Rotavirus C Replication in Porcine Intestinal Enteroids Reveals Roles for Cellular Cholesterol and Sialic Acids. Viruses.

[58] Ma P., Fang P., Ren T., Fang L., Xiao S. (2022). Porcine Intestinal Organoids: Overview of the State of the Art. Viruses.

[59] Yan M., Su A., Pavasutthipaisit S., Spriewald R., Graßl G.A., Beineke A., Hoeltig D., Herrler G., Becher P. (2023). Infection of porcine enteroids and 2D differentiated intestinal epithelial cells with rotavirus A to study cell tropism and polarized immune response. Emerg. Microbes Infect..

[60] Simsek C., Bloemen M., Jansen D., Descheemaeker P., Reynders M., Van Ranst M., Matthijnssens J. (2022). Rotavirus vaccine-derived cases in Belgium: Evidence for reversion of attenuating mutations and alternative causes of gastroenteritis. Vaccine.

[61] Maringa W.M., Simwaka J., Mwangi P.N., Mpabalwani E.M., Mwenda J.M., Mphahlele M.J., Seheri M.L., Nyaga M.M. (2021). Whole Genome Analysis of Human Rotaviruses Reveals Single Gene Reassortant Rotavirus Strains in Zambia. Viruses.

[62] Jiang S.Y., Zhao S.S., Wei J.Q., Zhang S., Zhao Z., Tong Y., Liu W., Wang J., Jiang T., Li J. (2025). General Intelligence Framework to Predict Virus Adaptation Based on a Genome Language Model. Research.

[63] Sinno A., Baghdadi R., Narch R., El Rayes S., Tokajian S., Al Khoury C. (2025). Charting the virosphere: Computational synergies of AI and bioinformatics in viral discovery and evolution. J. Virol..

[64] Hemming M., Vesikari T. (2012). Vaccine-derived human-bovine double reassortant rotavirus in infants with acute gastroenteritis. Pediatr. Infect. Dis. J..

